# Mesalazine allergy and an attempt at desensitization therapy in patients with inflammatory bowel disease

**DOI:** 10.1038/s41598-020-79207-z

**Published:** 2020-12-17

**Authors:** Satohiro Matsumoto, Hirosato Mashima

**Affiliations:** Department of Gastroenterology, Saitama Medical Center, Jichi Medical University, 1-847 Amanuma, Omiya, Saitama, Saitama 330-8503 Japan

**Keywords:** Gastroenterology, Gastrointestinal diseases, Inflammatory bowel disease

## Abstract

Mesalazine is a key drug used for remission induction and maintenance therapy in inflammatory bowel disease (IBD). We sometimes encounter patients who develop allergic reactions to the drug and inevitably discontinue treatment. Of 692 patients who received mesalazine for IBD between 2014 and March 2020, 33 diagnosed with mesalazine allergy (43 episodes) were included, and their clinical manifestations were evaluated. For ten patients undergoing desensitization therapy, therapeutic outcomes were evaluated. The incidence of mesalazine allergy was 4.8%. The time from the start of oral medication to allergy onset was 10 ± 5 days for the first allergic attack and 2 ± 1 days for the second and subsequent allergic attacks. The observed clinical symptoms included fever (93%), diarrhea (26%), abdominal pain (23%), and bloody stool (12%). Drug-induced lymphocyte stimulation test was performed in 85% of the patients (28/33), and the sensitivity was 51%. Desensitization therapy with a time-dependent mesalazine granule formulation was successful in nine of the ten patients (90%), allowing them to receive 2000 mg or more of the drug. Fever was a common allergic symptom, and its presence appeared to be useful for distinguishing mesalazine allergy from exacerbation of the primary disease. Desensitization therapy was useful in patients with mesalazine allergy.

## Introduction

Mesalazine is a key drug used for the treatment of ulcerative colitis. However, mesalazine sometimes exacerbates symptoms such as fever, diarrhea, and bloody stool. Moreover, some patients develop mesalazine intolerance, which is difficult to distinguish from exacerbation of the primary disease^1,2^. Adverse reactions to mesalazine are observed in 15.8% of patients with ulcerative colitis. Of these patients, 4.6% present with diarrhea and 1.4% present with bloody stool or fever^1^. Our previous report showed that 5.5% of patients with ulcerative colitis developed adverse reactions to pH-dependent mesalazine formulations^3^. Drug intolerance is a pharmacological reaction that does not represent any abnormalities in the metabolism, clearance, or bioavailability of drugs but evokes adverse reactions without causing immunoreactions of the humoral or cellular immune mechanism. In contrast, drug allergy is an immunologically mediated reaction to pharmacologically active agents or inactive ingredients. It occurs after the sensitization phase, and its development is associated with drug-specific antibodies, sensitized T cells, or both^4^. Although we have come across cases in which patients develop allergic reactions to mesalazine and inevitably discontinue the drug, reports of such cases are limited. Upon the onset of mesalazine allergy, because patients become reluctant to take mesalazine, the initiation of treatment with immunomodulators or biological agents must be considered as the next step of treatment. The penetrance of biologic use of inflammatory bowel disease has steadily risen. Biological agents are extremely expensive as compared with conventional medicines, and thus the economic impact of biological agents on healthcare is becoming a problem in recent years^5^. Immunomodulators are associated with serious risks of infections and certain malignancies^6,7^. Therefore, we hesitant to use these agents in patients that can be controlled with mesalazine. In addition, there are reports on desensitization therapy with mesalazine for patients with mesalazine intolerance^1^. If desensitization therapy is successful, treatment escalation to immunomodulators or biological agents will be unnecessary, and mesalazine therapy can be continued. Thus, we examined patients who developed mesalazine allergy, and desensitization therapy was attempted in some of them.

## Methods and materials

### Subjects

Of 692 Japanese patients with inflammatory bowel disease who started mesalazine therapy between 2014 and March 2020 and were followed-up at the outpatient clinic of Saitama Medical Center (538 with ulcerative colitis and 154 with Crohn's disease), 33 patients with 43 episodes who were diagnosed with mesalazine allergy (17 men and 16 women; disease duration, 0.8 ± 3.0 years) were included. According to the mesalazine formulations, 309 patients were prescribed a pH-dependent mesalazine formulation (ASACOL, Zeria Pharmaceutical, Tokyo, Japan), 131 were prescribed a pH-dependent mesalazine formulation with a multi-matrix system (LIALDA, Mochida Pharmaceutical, Tokyo, Japan), 460 were prescribed a time-dependent mesalazine formulation (PENTASA, Kyorin Pharmaceutical, Tokyo, Japan), and 58 were prescribed salazosulfapyridine (SLAZOPYRIN, Pfizer Pharmaceutical, Tokyo, Japan). These patients included those who had been previously treated with multiple mesalazine formulations. Patients were divided into two groups based on the presence of mesalazine allergy and retrospectively analyzed. Subsequently, clinical courses were assessed in the mesalazine allergy group. All data were collected exclusively by reviewing preexisting medical records.

### Diagnosis of mesalazine allergy

In previous reports, mesalazine intolerance was defined as a case in which any one of the following symptoms occurs after the introduction of mesalazine therapy: headache, gastrointestinal symptoms, skin manifestations, and fever^8–10^. In the present study, mesalazine allergy was defined as a case in which symptoms, such as fever, abdominal pain, diarrhea, and bloody stool, appeared or worsened after the introduction of mesalazine therapy and were immediately relieved after the discontinuation of oral mesalazine administration.

### Drug-induced lymphocyte stimulation test

Drug-induced lymphocyte stimulation test (DLST) (SRL, Inc., Tokyo, Japan) is a test method to determine the degree of division/proliferation of lymphocytes by measuring the uptake level of ^3^H-thymidine in a mixture of a suspected drug and sensitized lymphocytes obtained from patients suspected to have drug allergy. A 12-mL blood sample per tablet or capsule was collected from each patient and transferred to heparinized blood collection tubes. Each additional tablet or capsule required 5.0 mL of blood. The ratio of measurements in mixtures with and without the drug was expressed as stimulation index (SI), and patients with SI of > 180% were diagnosed as DLST-positive.

### Mesalazine desensitization therapy

A time-dependent mesalazine granule formulation (PENTASA) was used for mesalazine desensitization therapy. We developed a new protocol by referring to the protocol of Oustamanolakis et al.^11^. The regimen is shown in Table 1. For patients who received a 2000-mg dose without showing allergic reactions, the dose was increased up to 4000 mg according to their disease conditions at each step of desensitization therapy.

**Table 1:** Desensitization protocol with time dependent-mesalazine granules.

Day	Dose (mg)	Day	Dose (mg)	Day	Dose (mg)
1	50	11	550	21	1100
2	100	12	600	22	1200
3	150	13	650	23	1300
4	200	14	700	24	1400
5	250	15	750	25	1500
6	300	16	800	26	1600
7	350	17	850	27	1700
8	400	18	900	28	1800
9	450	19	950	29	1900
10	500	20	1000	30	2000

### Ethical considerations

This study was conducted with the approval of the Etiological Study Ethical Review Board of Saitama Medical Center, Jichi Medical University (S19-091). Because we produced anonymized data for use in this study, it was deemed not necessary to obtain informed consent from the study subjects. The need for the informed consent was waived by the research ethics committee.

The clinical procedures were carried out in accordance with the Declaration of Helsinki.

### Statistical analysis

Data are expressed as means ± SD or percentages. Statistical analyses were performed using the Student’s t-test and Fisher’s exact test. All data analyses were performed using the StatView software (version 5.0; SAS Institute Inc., Cary, NC, USA). Differences at *P* values of less than 0.05 were regarded as being significant.

## Results

Patient characteristics are shown in Table 2. No significant differences were observed between the allergy and non-allergy groups in sex, disease type, age at onset, each disease location, or type of mesalazine. Disease duration was 0.8 ± 3.0 years in the allergy group and 7.4 ± 8.4 years in the non-allergy group (*P* < 0.001). The dose of mesalazine at the start of treatment was 3640 ± 1010 mg in the allergy group and 2820 ± 1240 mg in the non-allergy group (*P* < 0.001). C-reactive protein (CRP) level at the start of treatment was 1.8 ± 2.8 mg/dL in the allergy group and 1.2 ± 2.5 mg/dL in the non-allergy group, with no significant differences between the two groups.

**Table 2:** Baseline characteristics.

	Allergy group (n = 33)	Non-allergy group (n = 659)	*P* value
Sex, male/female	17/16	402/257	0.28
Age at onset (years)	32 ± 14 (14–67)	36 ± 16 (5–81)	0.21
IBD type, UC/CD	30/3	508/151	0.08
Duration of disease (years)	0.8 ± 3.0 (0.0–19.8)	7.4 ± 8.4 (0.0–53.3)	< 0.001
Extent of UC, E1/E2/E3	2/6/22	73/132/303	0.34
Disease location of CD, L1/L2/L3	0/0/3	50/40/61	0.19
**Type of mesalazine**			0.13
Time dependent-release mesalazine	16 (37.2%)	444 (48.5%)	
pH dependent-release mesalazine	14 (32.6%)	295 (32.2%)	
Multimatrix mesalazine	11 (25.6%)	120 (13.1%)	
Salazosulfapyridine	2 (4.7%)	56 (6.1%)	
Dose of mesalazine (mg)	3640 ± 1010 (1000–4800)	2820 ± 1240 (500–6000)	< 0.001
CRP at the start of mesalazine (mg/dL)	1.8 ± 2.8 (0.03–8.70)	1.2 ± 2.5 (0.01–18.74)	0.26

Disease extent in UC is classified as proctitis [E1], left-sided [E2] or extensive colitis [E3]. Disease location in CD is classified as ileum [L1], colon [L2] or ileocolon [L3].

*IBD* inflammatory bowel disease, *UC* ulcerative colitis, *CD* Crohn’s disease, *CRP* C-reactive protein.

The incidence of mesalazine allergy was 4.8% (33/692). According to the mesalazine formulations, the incidence was 4.5% (14/309) for the pH-dependent mesalazine formulation (ASACOL), 8.4% (11/131) for the pH-dependent mesalazine formulation with a multi-matrix system (LIALDA), 3.5% (16/460) for the time-dependent mesalazine formulation (PENTASA), and 3.4% (2/58) for salazosulfapyridine (SLAZOPYRIN). Of the 43 episodes, three were caused by topical mesalazine. At the onset of allergic reactions, concomitant drugs were administered in 32.6% of all episodes. Prednisolone was used in 27.9% (12/43), whereas adalimumab, tacrolimus, and histamine H2 blockers were used in 2.3% (1/43) each. The time from the start of oral medication to the onset of allergy was 9.8 ± 5.1 days (1–21 days) for the first allergic attack and 2.2 ± 1.1 days (1–5 days) for the second and subsequent allergic attacks. The observed clinical symptoms included fever in 93.0% of the episodes (40/43), diarrhea in 25.6% (11/43), abdominal pain in 23.3% (10/43), and bloody stool in 11.6% (5/43). No difference was found between the clinical symptoms or severity of symptoms in the first allergic attack and second and subsequent allergic attacks. At the onset, CRP level was 5.3 ± 5.3 mg/dL, white blood cell count was 9390 ± 2900/μL, and eosinophil count was 2.0 ± 1.8%. The symptoms were alleviated within 1.7 ± 0.7 days (1–3 days) after discontinuation of the drug. (Table 3).

**Table 3:** Clinical course of 43 mesalazine allergy cases (n = 33).

**Mesalazine allergy rate**^**a**^	4.8% (33/692)
Time dependent-release mesalazine	3.5% (16/460)
pH dependent-release mesalazine	4.5% (14/309)
Multimatrix mesalazine	8.4% (11/131)
Salazosulfapyridine	3.4% (2/58)
**Type of symptom (n = 43)**	
Fever	40 (93.0%)
Diarrhea	11 (25.6%)
Abdominal pain	10 (23.3%)
Hematochezia	5 (11.6%)
**Period from initiation of mesalazine to allergy onset (days)**	
First reaction	9.8 ± 5.1 (1–21)
Second or third reaction	2.2 ± 1.1 (1–5)
**Period from discontinuation of mesalazine to disappearance of symptom (days)**	1.7 ± 0.7 (1–3)
**Hematological examination**	
WBC (/μL)	9390 ± 2900 (3250–15,870)
Eosinophil (%)	2.0 ± 1.8 (0–6)
CRP (mg/dL)	5.3 ± 5.3 (0.02–20.03)
**Concomitant treatment (n = 43)**	14 (32.6%)
Prednisolone	12 (27.9%)
Adalimumab	1 (2.3%)
Tacrolimus	1 (2.3%)
H2 blocker	1 (2.3%)

*WBC* white blood cell, *CRP* C-reactive protein.

^**a**^The allergy incidence rate for each drug includes cases with a history of taking multiple types of mesalazine.

DLST was performed in 85% of the patients (28/33), with a sensitivity of 51%. The time from allergy onset to DLST was 7.8 ± 9.4 days (0–33 days) in 19 DLST-positive patients for the suspected drug, but 2.5 ± 1.6 days (0–5 days) in 18 DLST-negative patients.

**Figure 1:**
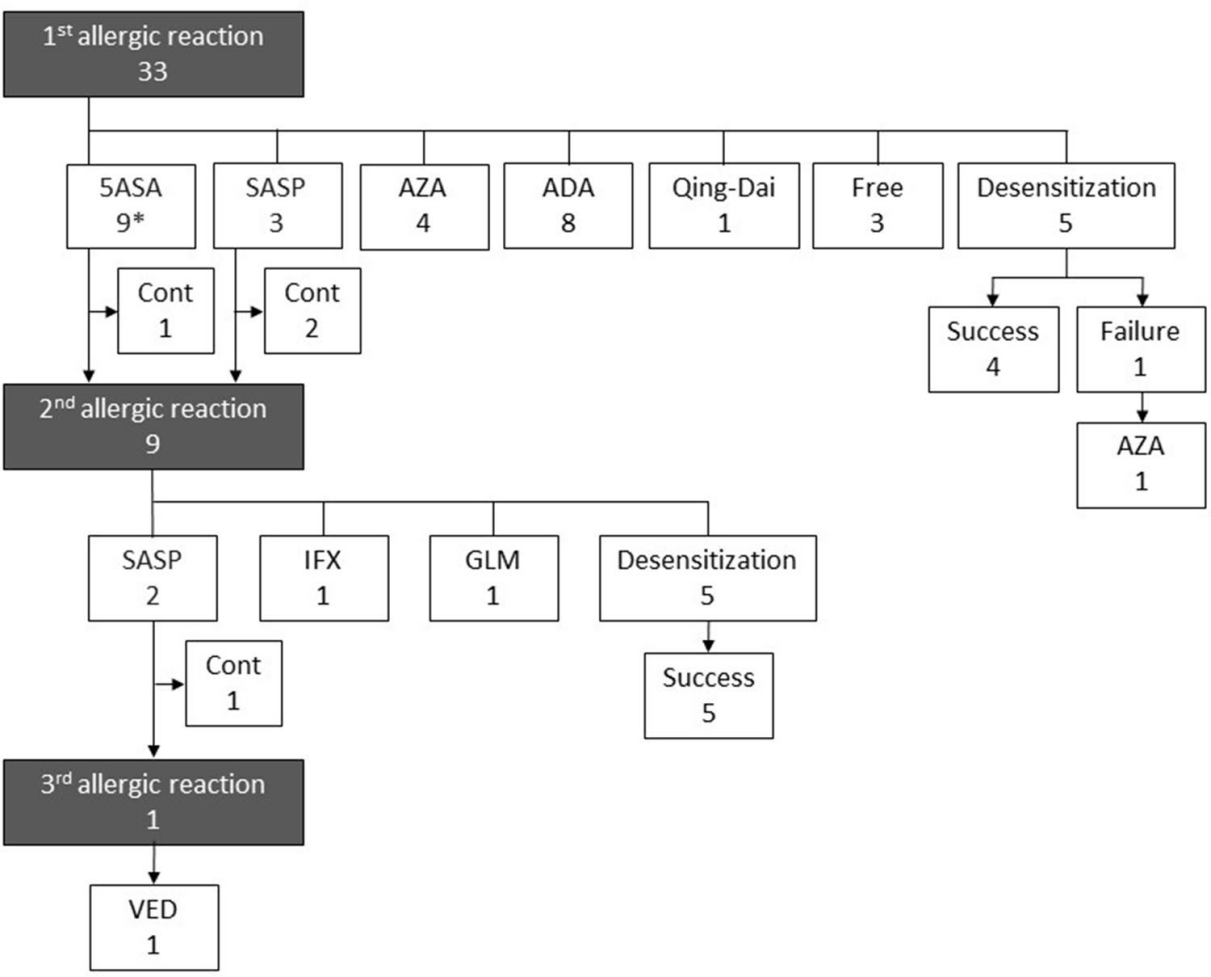
Flowchart of outcomes of mesalazine allergy patients. *Of nine patients who received other mesalazine formulations, three received multi-matrix mesalazine (4800 mg in all three patients), three received pH-dependent mesalazine (3600 mg in all three patients), and three patients received time-dependent mesalazine (4000 mg, 3000 mg, and 1000 mg). *5ASA* 5-Aminosalicylic acid, *SASP* salazosulfapyridine, *AZA* azathioprine, *ADA* adalimumab, *IFX* infliximab, *GLM* golimumab, *VED* vedolizumab, *Cont* continued.

The clinical course of the patients is shown in Fig. 1. Of 33 patients with the first allergic attack, 12 received other mesalazine formulations or salazosulfapyridine for which DLST results were negative; of these, 75% (9/12) developed a second allergic attack. In two of the nine patients with the second allergic attack, the medications were switched to salazosulfapyridine, for which the DLST results were negative. One patient did not develop a third allergic attack, but the other did. Because the latter was intolerant to azathioprine, vedolizumab therapy was ultimately introduced. Desensitization therapy with a time-dependent mesalazine granule formulation (PENTASA) was performed in ten patients with relatively mild symptoms. It was successful in nine patients (90%), who were then able to ingest 2000 mg or more of the drug. Thereafter all patients achieved clinical remission without exacerbation during desensitization therapy. In nine of the ten patients who received desensitization therapy, DLST was performed for PENTASA. The results were positive in four patients and negative in five patients. The patient in whom desensitization therapy was unsuccessful showed a positive DLST result and was ultimately treated with azathioprine (Table 4).

**Table 4:** Clinical course of ten patients that underwent desensitization protocol with time dependent-mesalazine granules.

	IBD type	Location	Sex	Age	DLST for PENTASA	Maximal doses (mg)	Clinical score before desensitization	Clinical score 3 m after desensitization	Efficacy of desensitization	Observation period from the time to reach final maximal doses (days)
1	UC	E3	M	56	Positive	2000	3	2	Success	35
2	UC	E2	M	28	Negative	2000	3	2	Success	38
3	UC	E2	F	50	Negative	4000	6	1	Success	375
4	UC	E3	F	24	Negative	3000	4	2	Success	53
5	UC	E2	M	17	Negative	3500	4	2	Success	267
6	UC	E3	F	15	Positive	NA	6	NA	Failure	NA
7	UC	E2	F	23	Negative	2000	4	2	Success	32
8	UC	E2	F	65	Positive	2000	4	2	Success	35
9	UC	E3	F	47	Positive	4000	4	3	Success	52
10	CD	L3	M	14	–	2000	148	46	Success	32

Disease extent in UC is classified as proctitis [E1], left-sided [E2] or extensive colitis [E3].

Disease location in CD is classified as ileum [L1], colon [L2] or ileocolon [L3].

## Discussion

Mesalazine is a first-line drug for the treatment of ulcerative colitis. It is important to deliver a sufficient amount of the drug to the large intestine and maintain its high concentration in the intestinal mucosa for mucosal healing. At present, the available oral mesalazine formulations in Japan are salazosulfapyridine (SLAZOPYRIN), a time-dependent mesalazine formulation (PENTASA), and pH-dependent mesalazine formulations (ASACOL and LIALDA). However, mesalazine may cause and exacerbate symptoms, such as fever, abdominal pain, diarrhea, and bloody stool. This causes difficulties in distinguishing mesalazine-induced symptoms from exacerbated symptoms of ulcerative colitis. The incidence of mesalazine intolerance in ulcerative colitis varies from 2.1 to 24% among reports^12–14^.

The allergic symptoms observed in the present study were fever (93%), diarrhea (26%), abdominal pain (23%), and bloody stool (12%). Fever was common, and all patients with the symptom had high fever of ≥ 38 °C. It has been reported that, at the onset, the CRP level is elevated to 5.3 mg/dL, and mesalazine allergy is reportedly characterized by the absence of eosinophilia^12^. Consistent with this previous finding, in the present study, the mean CRP level was 5.1 mg/dL, and eosinophilia was not observed. Although drug allergy is generally dose-independent^15^, in this study, the dose of mesalazine at the start of treatment in the allergy group was significantly higher than that in the non-allergy group. This may be because the non-allergic group contained more patients with Crohn’s disease, for which the maximum dose of mesalazine is lower than that for ulcerative colitis, and ulcerative proctitis, in which topical formulation is the first choice, than the allergic group. The allergic group contained more patients treated with a pH-dependent mesalazine formulation with a multi-matrix system with the highest upper limit than the non-allergic group. In addition, the time interval to allergy onset is commonly within 2 weeks^4^; in the present study, the first allergic attack occurred at 10 days after medication, and the second attack occurred at 2 days after medication. Mesalazine allergy appears to be a type IV allergy, in which inflammation is caused by reactions between antigens and T cells recognizing antigens (especially T helper 1 cells). The development of type IV allergy occurs in two phases: the sensitization and elicitation phases. Upon the initial invasion of antigens, they are internalized by antigen-presenting cells, which activate T cells in regional lymph nodes. In this process, memory T-cells are generated along with effector T-cells, and they promptly respond to the second and subsequent invasion (the sensitization phase). In the event of the second or subsequent invasion, the antigen-presenting cells activate the memory T-cells, which elicit inflammation peaking at 48 h (the elicitation phase). In the present study, the first allergic attack occurred several days after the start of administration of the responsible agent, and the second allergic attack occurred at 2 days after the start. These findings indicate that mesalazine allergy was a type IV allergy.

DLST is sometimes performed as a supplemental test for the diagnosis of mesalazine allergy. A report on drug-induced liver injury in Japan showed that the positive rate of DLST is 33%^16^. In patients with ulcerative colitis, DLST has been reported to have a sensitivity of 24.0% and a specificity of 80.5%^11^. Because DLST has low sensitivity and high specificity in patients suspected to have mesalazine allergy, making a definitive diagnosis of mesalazine allergy using DLST instead of through exclusion appears to be appropriate. There are also a certain number of false negative cases^13^. In fact, a diagnosis of allergy should be based on the evaluation of the clinical course. Furthermore, attention should be paid to the timing of performing DLST and the use of concomitant drugs. Regarding the timing of performing DLST, the time from allergy onset to blood sample collection in the present study was significantly longer in patients with positive DLST for the suspected drug than in patients with negative DLST for the suspected drug. DLST is based on the reactions of memory T-cells. When hypersensitive reactions are occurring, memory T-cells are eccentrically located and regulatory T-cells are strongly activated. In these conditions, the test sometimes yield false negative results. Thus, it is considered that DLST should be performed after remission or at 4 weeks after allergic reactions^17,18^. Regarding concomitant drugs, steroids may mask allergic symptoms. If the worsening of symptoms is judged to be exacerbation of the primary disease and leads to immediate addition of steroids, the symptoms will be masked. While steroids are tapered, the symptoms will relapse. Therefore, the patient may be judged to be steroid-dependent, which may result in overtreatment or a delayed diagnosis of mesalazine allergy^19^. In addition, we previously experienced a case of mesalazine allergy that was difficult to diagnose because of adalimumab^20^. Although DLST detects type IV allergy associated with T-lymphocytes, adalimumab also inhibits T-lymphocytes. Thus, it appears that adalimumab might have masked mesalazine allergy and alleviated the symptoms.

In the present study, 9 of 33 patients with the first allergic attack received other mesalazine formulations for which DLST results were negative, and eight patients (89%) developed a second allergic attack. In two of the nine patients with the second allergic attack, their medications were switched to salazosulfapyridine, for which DLST results were negative. One patient (50%) developed the third allergic attack. Regarding the treatment strategy after the first allergic attack, if a patient’s disease activity was mild to moderate, we presented other mesalazine formulations or salazosulfapyridine for which DLST results were negative, or desensitization therapy as treatment options. In the present study, because of the low sensitivity of DLST, only 23% of the patients ultimately switched their medications to other mesalazine formulations for which DLST results were negative. Symptoms were alleviated within 2 days after discontinuation of the responsible agent, and no patient exhibited serious symptoms. Thus, an attempt to switch to another mesalazine formulation is fully acceptable. However, drug allergy may sometimes cause serious adverse reactions. For this reason, desensitization therapy with mesalazine is expected to be an effective therapeutic option particularly for patients with mild symptoms, who have developed mesalazine allergy.

Many reports indicated that the success rate of desensitization therapy with salazosulfapyridine is more than 80% in patients intolerant to salazosulfapyridine^21–24^, and recent reports have shown that desensitization therapy with mesalazine is effective for mesalazine intolerance, yielding relatively good outcomes^1,14,25,26^. In the present study, desensitization therapy with a time-dependent mesalazine granule formulation (PENTASA) was performed in ten patients. It was successful in nine patients, with a success rate of 90%. Among the mesalazine formulations available in Japan, only PENTASA is a granule formulation. The use of this formulation is preferable for desensitization therapy in which the dose is gradually increased. Although there are reports of desensitization therapy performed in an inpatient setting for a short period of time^25–27^, no consensus has been reached on incremental dosing regimens, which remain controversial. Regarding the precautions to be taken while initiating desensitization therapy, we consider that desensitization therapy should not be performed in patients with organ disorders, such as agranulocytosis, liver disorders, and lung disorders.

The study limitations include the single-center retrospective cohort study design and the small sample size. However, it is not easy to enroll the large number of IBD patients with mesalazine allergy. This study will stimulate further research on desensitization for mesalazine allergy.

In conclusion, a common symptom of mesalazine allergy was fever, and its presence appeared to be useful for distinguishing mesalazine allergy from exacerbation of the primary disease. Because the timing of onset varied between the first allergic attack and the second or subsequent attack, an explanation about this should be provided to patients. Desensitization therapy was shown to be useful for patients with mesalazine allergy and appeared to be a therapeutic strategy worth trying before treatment escalation to the next stage.
